# Whole genome phylogenetic investigation of a West Nile virus strain isolated from a tick sampled from livestock in north eastern Kenya

**DOI:** 10.1186/s13071-014-0542-2

**Published:** 2014-11-28

**Authors:** Olivia Wesula Lwande, Marietjie Venter, Joel Lutomiah, George Michuki, Cecilia Rumberia, Francis Gakuya, Vincent Obanda, Caroline Tigoi, Collins Odhiambo, Fredrick Nindo, Samwel Symekher, Rosemary Sang

**Affiliations:** International Centre of Insect Physiology and Ecology, Nairobi, Kenya; Department of Medical Virology, University of Pretoria, Pretoria, South Africa; Kenya Medical Research Institute, Nairobi, Kenya; Kenya Wildlife Service, Nairobi, Kenya; International Livestock Research Institute, Nairobi, Kenya; Computational Biology Group, Institute of Infectious Diseases and Molecular Medicine, (IIDMM) University of Cape Town, Cape Town, South Africa; Global Disease Detection, United States-Centers for Disease Control, Pretoria, South Africa

**Keywords:** West Nile virus, Tick, Kenya, Livestock, Wildlife

## Abstract

**Background:**

West Nile virus (WNV) has a wide geographical distribution and has been associated to cause neurological disease in humans and horses. Mosquitoes are the traditional vectors for WNV; however, the virus has also been isolated from tick species in North Africa and Europe which could be a means of introduction and spread of the virus over long distances through migratory birds. Although WNV has been isolated in mosquitoes in Kenya, paucity of genetic and pathogenicity data exists. We previously reported the isolation of WNV from ticks collected from livestock and wildlife in Ijara District of Kenya, a hotspot for arbovirus activity. Here we report the full genome sequence and phylogenetic investigation of their origin and relation to strains from other regions.

**Methods:**

A total of 10,488 ticks were sampled from animal hosts, classified to species and processed in pools of up to eight ticks per pool. Virus screening was performed by cell culture, RT-PCR and sequencing. Phylogenetic analysis was carried out to determine the evolutionary relationships of our isolate.

**Results:**

Among other viruses, WNV was isolated from a pool of *Rhipicephalus pulchellus* sampled from cattle*,* sequenced and submitted to GenBank (Accession number: KC243146). Comparative analysis with 27 different strains revealed that our isolate belongs to lineage 1 and clustered relatively closely to isolates from North Africa and Europe, Russia and the United States. Overall, Bayesian analysis based on nucleotide sequences showed that lineage 1 strains including the Kenyan strain had diverged 200 years ago from lineage 2 strains of southern Africa. Ijara strain collected from a tick sampled on livestock was closest to another Kenyan strain and had diverged 20 years ago from strains detected in Morocco and Europe and 30 years ago from strains identified in the USA.

**Conclusion:**

To our knowledge, this is the first characterized WNV strain isolated from *R. pulchellus*. The epidemiological role of this tick in WNV transmission and dissemination remains equivocal but presents tick verses mosquito virus transmission has been neglected. Genetic data of this strain suggest that lineage 1 strains from Africa could be dispersed through tick vectors by wild migratory birds to Europe and beyond.

**Electronic supplementary material:**

The online version of this article (doi:10.1186/s13071-014-0542-2) contains supplementary material, which is available to authorized users.

## Background

West Nile virus (WNV) is classified in the family *Flaviviridae*, genus *Flavivirus* which is closely related to viruses such as Japanese encephalitis, Saint Louis encephalitis, Usutu, Kunjin, Kokobera, Stratford, Alfuy and Murray Valley encephalitis that belong to the Japanese encephalitis serocomplex [1]. WNV has a wide geographical distribution mainly in Africa, Europe, Russia, the Middle East, India, Australia and North and South America and the Caribbean [2-4]. Recent outbreaks of WNV have been reported in Israel, France, Italy, Greece, South Africa, Hungary, southeast Romania and the USA [5-10].

WNV is known to be transmitted primarily by mosquitoes of the genus *Culex* (principally *C. univittatus* and *C. pipiens*) [11-14] although evidence of tick-borne transmission has also been documented [15,16]. Vector competence studies performed mainly on soft ticks such as *Argas persicus*, *A. hermanni* [17] and *Ornithodoros moubata* [15] indicate the potential role of ticks in WNV transmission. Although vector competence studies indicate a potential role for ticks in WNV transmission, results of these studies indicate that ticks are likely to be far less efficient vectors of WNV than mosquitoes [15]. The ability of WNV to replicate in mosquitoes, mammals and birds provides an opportunity for this virus to amplify across a wider geographical coverage enabling the circulation of different strains across continents.

WNV causes mainly mild febrile illness but can result in meningo-encephalitis, acute paralysis and death in severe cases in humans and horses [7,18-22]. In humans, the proportion of elderly patients presenting with severe neurologic illness due to WNV has been reported to be high in the USA [23,24].

WNV genome is composed of an 11 kb positive RNA fragment that is translated to form a polyprotein. This polyprotein consists of three structural (C, prM/M and E) and seven non-structural proteins (NS1, NS2A, NS2B, NS3, NS4A, NS4B and NS5) [25]. Phylogenetically, WNV belongs to two major lineages, Lineage 1 and 2. Lineage 1 is composed of three sub-lineages a, b and c. Lineage 1a is distributed in Africa, the Middle East, Europe and America. Lineage 1b, which is also known as Kunjin virus (KUNV), is found in Australia and Linegae 1c mainly circulates in India. WNV Lineage 2 comprises strains isolated in sub-Saharan Africa [26]. Other proposed WNV lineages include Lineage 3, also known as Rabensberg virus, that occurs in southern Moravia and Czech Republic, Lineage 4 from Russia, Lineage 5 from India and Lineage 6 that is also known as Koutango virus [27-29].

Previous studies indicate that WNV circulates in Kenya, having been isolated from diverse mosquito species [30-33]. Evidence of natural isolation of WNV from *Rh. pulchellus* ticks sampled from cattle and warthog has been documented in Kenya [34], although there is scanty genetic and pathogenicity data on this virus since it has not been characterized in Kenya. In addition, the genetic profile and phylogeny of the circulating strains remains unresolved. Therefore the objectives of this study were (i) to determine the role of ticks in the maintenance and circulation of arboviruses in Kenya and (ii) to genetically analyze the isolated strains. This study was carried out during 2010–2012 in Ijara district, a semi-arid pastoral region which periodically experiences RVFV outbreaks that tend to co-circulate with other mosquito-borne arboviruses like WNV.

This study aimed to investigate circulation, transmission and diversity of WNV in Ijara District and test the hypothesis of its spread through migratory birds and tick vectors across the continent. This study will also aid in assessing the risk of disease, monitoring emerging tick-borne viral diseases and providing a better understanding of the patterns and processes of evolution.

## Methods

This was a field-based descriptive cross-sectional and laboratory-based study conducted between 2010 and 2012.

### Ethics statement

This study was approved by the Kenya Medical Research Institute Ethical Review Committee protocol number SSC 2050. The Committee of the Department of Veterinary and Capture Services (DVCS) of the Kenya Wildlife Service (KWS) approved the study including animal immobilization capturing protocols. KWS guidelines on Wildlife Veterinary Practice-2006 were followed. All KWS veterinarians were guided by the Veterinary Surgeons Act Cap 366 Laws of Kenya that regulates veterinary practices in Kenya. For purposes of livestock use, permission was obtained from the owners involved. We worked in collaboration with the department of veterinary services and veterinarians mandated by the government to do livestock sampling and research. The above terms were stipulated well in an agreement between the farmers and the International Centre of Insect Physiology and Ecology (*icipe*), the hosting institution for the Arbovirus Incidence and Diversity (AVID) Project Consortium.

### Study area

This study was carried out in Ijara District, North Eastern Province of Kenya. The district lies between latitude 1°7′S and 2°3′S and longitude 40°4′E and 41°32′E. This is a low altitude (ranging between 0 and 90 meters above sea level) arid and semi-arid region where 90% of the people practice nomadic pastoralism, keeping indigenous cattle, goats, sheep, donkeys and camels. Approximately one-quarter of the district is covered by the Boni forest bordering the Indian ocean, which is an indigenous open canopy forest that forms part of the Northern Zanzibar-Inhamdare coastal forest mosaic. A section of the forest, the Boni National Reserve is under the management of the Kenya Wildlife Service as a protected conservation area and is home to a range of wildlife species, including hirola antelope (also known as Hunter’s hartebeest), reticulated giraffe, elephant, buffalo, lion, leopard, cheetah, African wild dog, lesser kudu, desert warthog and bushbuck.

### Sampling of ticks

Ticks sampling from both domestic animals and wildlife was undertaken at various sites of the Ijara District, including the Boni National Game Reserve as described previously in [34]. Qualified animal handlers who wore the necessary protective gear (such as gloves, coveralls with trouser cuffs taped to shoes, high-top shoes, socks pulled over trouser cuffs, and long-sleeved shirts) performed tick collections. Livestock (goats, sheep and cattle) were physically restrained, whereas Kenya Wildlife Service veterinarians immobilized the wild animals (giraffe, warthog, lesser kudu and zebra) using a combination of etorphine hydrochloride (M99R, Novartis, South Africa) and xylazine hydrochloride (Kyron, South Africa). Both livestock and wild animals were visually examined for ticks, with special attention to the abdomen, back, anal area, and hind legs. If found, the ticks were pulled off manually, placed in sterile plastic vials, and transported to the laboratory in dry ice.

### Tick identification and processing

Tick identification and processing was performed as described previously in [34]. The ticks were washed twice with sterile water to remove excess particulate contamination from animal skin, rinsed once with 70% ethanol, and then rinsed twice with minimum essential medium (MEM) containing antimicrobial agents (100U/mL penicillin, 100 lg/mL streptomycin and 1 *μ*L/mL amphotericin B). Tick identification was performed using appropriate identification keys. The ticks were transferred to sterile vials and stored at -80°C until processed for virus isolation. Ticks were later thawed in ice (4°C), identified and pooled into groups of one to eight (depending on size) by species, sex, and animal host. Each pool was homogenized using 90-mesh alundum in a prechilled, sterile mortar and pestle with 1.6–2 mL ice-cold MEM containing 15% fetal bovine serum (FBS), 2% glutamine, 100U/mL penicillin, 100 lg/mL streptomycin, and 1 μL/mL amphotericin B. The homogenates were clarified by low-speed centrifugation at 1500 rpm for 15 min at 4°C, and supernatants aliquoted and stored at -80°C. In the case of *Hyalomma* spp., the primary vectors of Crimean–Congo hemorrhagic fever virus (CCHFV), each pool was prescreened for CCHF by reverse transcription-polymerase chain reaction (RT-PCR) to exclude this virus prior to cell culture screening.

### Virus isolation

Virus isolation was performed as described in [34]. Briefly, Vero cells were grown in 25-cm^2^ cell culture flasks to 80% confluency in MEM containing 10% FBS, 2% glutamine, 100U/mL penicillin, 100 lg/mL streptomycin, and 1 μ/mL amphotericin B. The cells were then rinsed with sterile phosphate-buffered saline (PBS) and 0.2 mL of clarified tick homogenate was added followed by incubation at 37°C for 45 min to allow virus adsorption. After incubation, MEM supplemented with 2% FBS, 2% glutamine, 100 U/mL penicillin, 100 lg/mL streptomycin, and 1 μ/mL amphotericin B was added into the flasks and the cells allowed to incubate at 37°C for 14 days while observing cytopathic effect on a daily basis. The supernatants of virus-infected Vero cell cultures exhibiting cytopathic effect of approximately 70% were harvested from the flasks for virus identification. The pooled infection rate program (PooledInfRat, Centers for Disease Control and Prevention, Fort Collins, CO; http://www.cdc.gov/ncidod/dvbid/westnile/software.htm/) was used to compare virus infection rates in the tick species collected and processed in this study.

### RNA sample preparation for 454 sequencing

Tick homogenates with positive cytopathic effect were filtered through a 0.22 μm filter, followed by RNA extraction using TRIzol reagent (Invitrogen). RNA was amplified using the modified random priming mediated sequence independent single primer amplification (RP-SISPA) methodology [35].

### 454 sequencing

Each amplified sample was further processed as described for shotgun library preparation in GS FLX 454 technology. The sequencing reads were trimmed to remove SISPA primers and barcodes and only reads with a length greater than 50 bp were retained. Low complexity repeats were masked using Repeatmasker (Repeat-Masker Open-3.0.1996-2010 http://www.repeatmasker.org) and sequences with more than 50% repeats were excluded. The sequences in each pool were assembled using the Newbler assembler version 2.5.3 with default settings (Roche. Genome Sequencer FLX Data Analysis Software Manual. Mannheim, Germany: Roche Applied Science, 2007). Contiguous sequences and reads which did not assemble into contigs were categorized using BLASTN and BLASTX homology searches against the non-redundant nucleotide and amino acid databases from NCBI (version June 2011). Taxonomic classification of each contig/read was investigated using MEGAN 4.0.

### Phylogenetic analysis

Comparative phylogenetic analyses were performed to describe and infer the lineage and occurrence of any selective pressures (amino acid substitutions) in the WNV strain isolated from *R. pulchellus* ticks in this survey in relation to strains isolated from other vectors (available in GenBank). The assembled sequence data isolated from ticks collected in Ijara District, Kenya were combined with available global full genome WNV reference sequences retrieved from the NCBI GenBank database. The final dataset comprised of 28 full genome sequences approximately 11,000 bp long. These were aligned using MUSCLE multiple sequence alignment program and manually edited and visualized using MacClade v4.08a [36]. The general time reversible (GTR) nucleotide substitution model with four gamma rate heterogeneity categories was found to be appropriate for these data based on the Modeltest analysis [37].

Phylogenies were obtained using RAxML v7.4.4 [38] implementing the GTR model of nucleotide substitutions with four gamma categories of rate heterogeneity, a method for phylogenetic tree inference using maximum likelihood/rapid bootstrapping techniques for long sequences. Statistical support for the clustering observed was assessed using bootstrapping techniques engraved in the RAxML algorithm.

To infer the evolutionary relationships among the selected WNV strains isolated from different vectors, dated ancestral states were reconstructed using BEAST available at (http://beast.bio.ed.ac.uk) under the GTR + gamma and proportion of invariant sites. BEAST employs Bayesian Markov Chain Monte Carlo MCMC tree estimation strategies. Two independent chain runs were conducted for 100 million generations, sampling trees and parameters at intervals of 10,000 generations under the uncorrelated lognormal relaxed clock model. To gauge the convergence of the runs, the log files were visualized in Tracer software included in the BEAST software package.

Clade support was assessed by calculating posterior probability for monophyletic clades observed in the tree topology. The trees obtained were summarised using Treeannotaor program included in BEAST software package with the first 10% trees obtained before the convergence of the runs discarded as “burn-in”.

### Amino acid substitution (selection) analysis

Comprehensive site-specific amino acid substitution analysis was done using SLAC and FUBAR methods available in Hyphy suite of selection pressure analysis tools accessible through data-monkey web server as (http://www.hyphy.org/w/index.php/SLAC and http://www.hyphy.org/w/index.php/FUBAR) for prediction of site specific amino acid changes and genome distribution of sites under positive and negative selection pressure, respectively [39,40]. The alignment was first analyzed for presence of any recombination using GARD program also embedded in the Hyphy and accessed through data-monkey web server graphical user interface [41]. Selection analyses were performed under the GTR model of nucleotide substitution and the significance of the prediction of the amino acids at specific sites were assessed statistically by p-values <0.1 and posterior probabilities ≥0.9 for SLAC and FUBAR analyses respectively [42].

## Results

A total of 10,488 ticks were sampled from both livestock and wild animal hosts and processed into 1,520 pools of up to eight ticks per pool. WNV screening in ticks collected from various animal hosts in Ijara District, Kenya, resulted in two tick-borne isolates obtained from *R. pulchellus* sampled from cattle and warthog as described in [34]. These isolates were confirmed to be WNV after being subjected to PCR testing and subsequent sequencing. These WNV isolates were subjected to Sanger sequencing of the non-structural protein 5 (NS5) gene region (positions 9254 to 9609) used for identification and confirmed to be WNV. One of the two isolates (ATH002316 GenBank accession number; KC243146.1) was subjected to 454 sequencing using random/shotgun sequencing to attempt full genome sequencing. Ninety-nine percent (99%) of the WNV genome was obtained by 454 sequencing. WNV genome positions 4 to 10,867 corresponding to polyprotein which consist of both structural (C, E and M) and non-structural (1, 2A, 2B, 3, 4A, 4B and 5) proteins and amino acid positions 1–3,434 was obtained.

### Phylogenetic clustering of the Kenyan Tick-borne WNV

**Figure 1:**
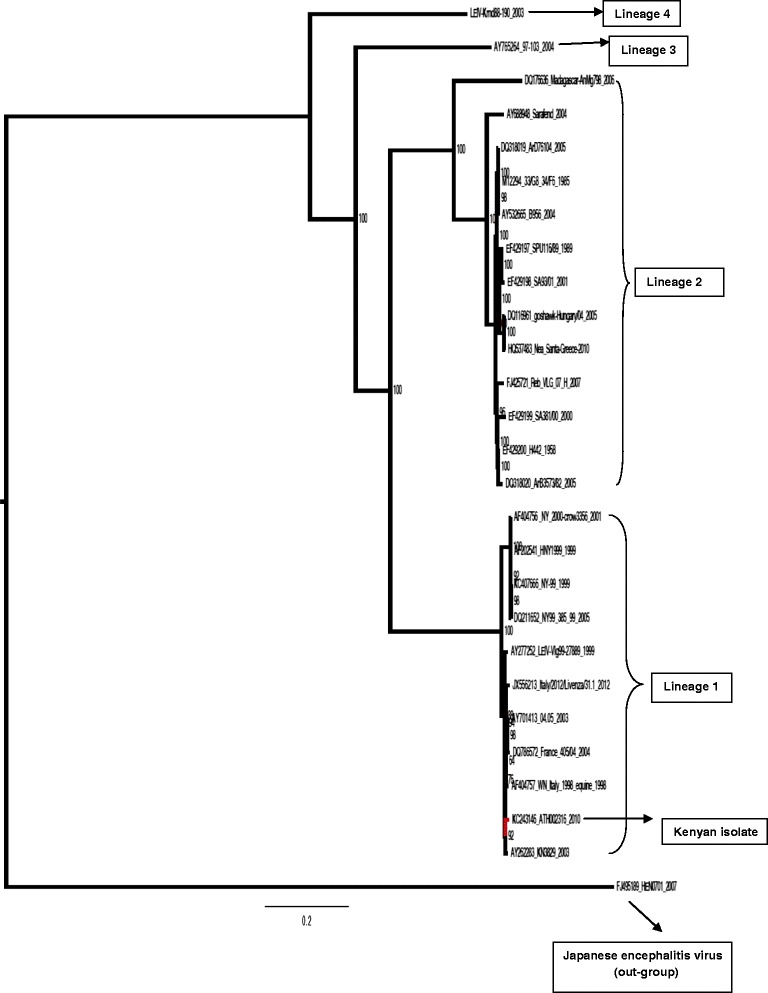
**Maximum likelihood phylogeny of selected WNV sequences.** GenBank accession numbers. Strain abbreviations (isolation source, country, year and accession number): SE-90: *Mimomyia lacustris*, Senegal, 1990, DQ318019; ATH002316, *R. pulchellus*, Kenya, 2010, KC243146.1; Ug-37: human, Uganda, 1937, AY532665; WNFCG: derivate of Ug-37, M12294; SA-89: human, South Africa, 1989, EF429197; SA-01: human, South Africa, 2001, EF429198; Hu-04: *Accipiter gentilis*, Hungary, 2004, DQ116961; Gr-10: *Culex pipiens*, Greece, 2010, HQ537483; SA-58: human, South Africa, 1958, EF429200; SA-00: human, South Africa, 2000, EF429199; Rus-07: human, Russia, 2007, FJ425721; Rab-97: *Cx. pipiens,* Czech Republic, 1997, AY765264.1; CAR-72: *Cx. tigripes,* Central African Republic, 1972, DQ318020.1; Sarafend: derivate of Ug-37, AY688948.1; *Coracopsis vasa*, Madagascar, 1978, DQ176636.2; Rus-98: *Dermacentor marginatus*, Russia, 1998, AY277252.1; human brain in 1999, LEIV-Vlg99-27889, Russia: Volgograd, low Volga, AY277252.1; NY-99, USA, KC407666.1; total brain RNA (patient NYC99002), HNY1999, USA: New York, AF202541.1; crow, WN NY 2000-crow3356, USA: New York, AF404756.1; NY99,385-99, USA, DQ211652.1; *Homo sapiens,* Italy/2012/Livenza/31.1, Italy: Veneto region, Venice province, JX556213.1; brain of horse with encephalitis, 2003, Morocco, AY701413.1; brain, house sparrow (Passer domesticus), France 405/04, France, 2004, DQ786572.1; equine, WN Italy 1998-equine, Italy, AF404757.1; *Culex univittatus,* KN3829, AY262283.1 and swine brain, China, 2007, FJ495189.1. Japanese encephalitis virus isolate GenBank accession number FJ495189.1 was used an out-group.

Twenty-six selected representative full or near full genome WNV sequences obtained from different vectors and corresponding clinical isolates from humans and animals from other parts of Africa, the Americas, Asia, Australia, and Europe were combined with the Kenyan isolate to make a final alignment of 27 sequences and used for phylogenetic analyses. Maximum likelihood phylogeny was obtained using RAxML program under GTR model of nucleotide substitution and bootstrapping with 1,000 replicates (Figure 1). The Kenyan strain (KC243146.1) clustered together with other strains from different parts of the world belonging to WNV Lineage 1.

### Evolutionary relationships among WNV strains

**Figure 2:**
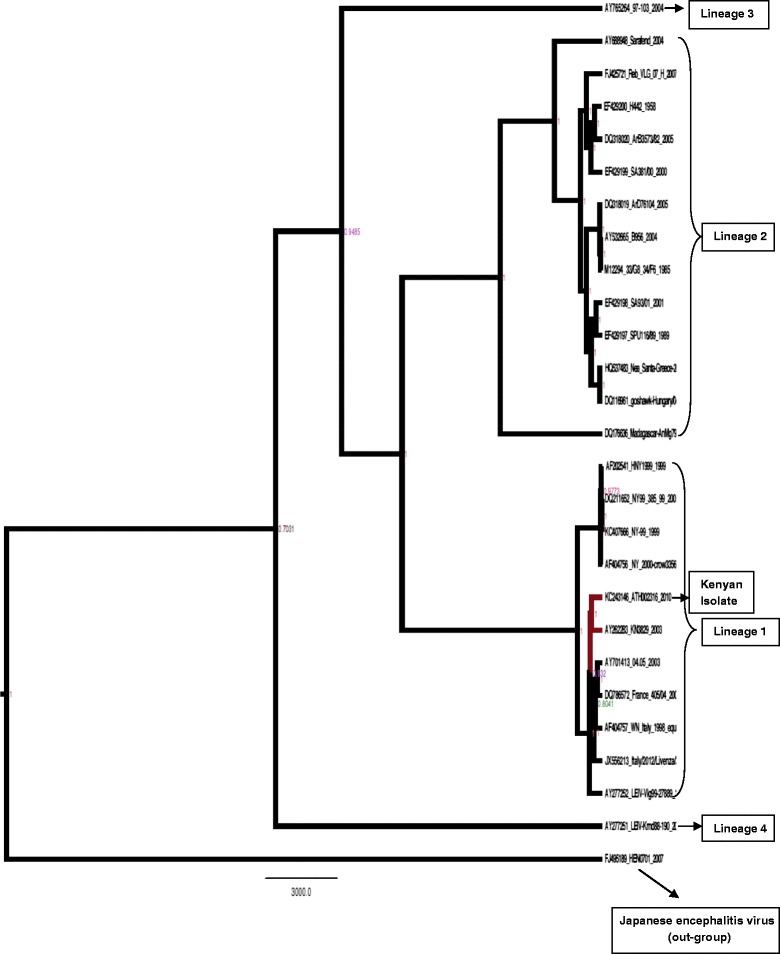
**Evolutionary relationships among the WNV strains.** Maximum Clade Credibility tree generated under GTR gamma model of nucleotide substitution with 4 gamma categories of rate heterogeneity and constant size coalescent population model assuming log-normal priors. GenBank accession numbers: DQ318019, KC243146.1, AY532665, M12294, EF429197, EF429198, DQ116961, HQ537483, EF429200, EF429199, FJ425721, AY765264.1, DQ318020.1, AY688948.1, DQ176636.2, AY277252.1, AY277252.1, KC407666.1, AF202541.1, AF404756.1, DQ211652.1, JX556213.1, AY701413.1, DQ786572.1, AF404757.1, AY262283.1 and FJ495189.1. Node labels are median heights (in years).

The evolutionary relationship of the Kenyan tick-borne WNV strain and other WNV strains was assessed using BEAST tool under general time reversible (GTR) model. The clustering observed under this method was similar to that observed under maximum likelihood providing further support that the WNV isolate (KC243146.1) from ticks collected in Ijara district in Northern Kenya clustered closely to Lineage 1 strains from Kenya as well as Europe, North Africa and the USA. The time to the most recent common ancestor of Lineage 1 viruses was estimated to be approximately 200 years (Figure 2) while the Ijara tick strain diverged approximately 21 years ago from the other Kenyan strain or European strains and 30 years ago from the USA strains. The strains from North Africa were closest to the strains studied in France and only diverged 9 years ago, suggesting this may have been the possible route of introduction to Europe. The Kenyan strain (KC243146.1) was distinct from Lineage 2 strains from southern Africa and diverged more than 200 years ago from these.

### Selection signatures (amino acid substitutions) in WNV genome

**Figure 3:**
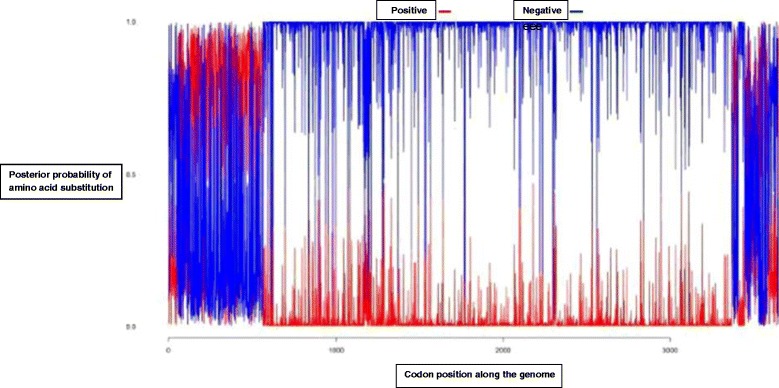
**Predicted codon site specific negative and positive selection (amino acid substitutions) along the WNV genome.** Sites under positive selection are indicated in red and sites predicted to be under negative selection are indicated in blue.

The 27 WNV full genome sequences were first tested for recombination. Two partitions of 1–980 codons and 981–3650 codon sites were obtained; however, no recombination signal was detected in any of the genomes used for these analyses. The Hyphy SLAC selection analysis method predicted 21 codon sites to be under positive selection based on significant p-values. (Figure 3). Site-specific amino acid changes as predicted from SLAC analysis in Hphy datamonkey webserver were extracted and recorded as shown in Additional file 1: Table S1.

To better visualize the distribution of sites under selective pressure, the distribution of predicted posterior probabilities for the respective codon sites along the WNV genome were plotted and it was observed that there was differential distribution of positive and negative pressures along the various genomic regions of WNV strains (Figure 3).

## Discussion

Studies conducted in Kenya during the RVF outbreaks demonstrated the presence of WNV in diverse mosquito species sampled from Rift Valley and North-Eastern Provinces. Although WNV has not been associated with any outbreak in Kenya, this virus has been isolated from male *C. univittatus* sampled from Rift Valley [33], *Aedes sudanensis* from North Eastern Kenya [32] and *Culex* spp. and *Aedes* spp. sampled from North Eastern Kenya [30]. Neutralizing antibodies against WNV have also been detected in sera of diverse mammalian species including cattle in Turkey, thus providing evidence of WNV circulation in the region [43]. The isolation of WNV from *R. pulchellus* sampled from cattle in Ijara District of North Eastern Province of Kenya suggests that this virus might be circulating amongst diverse vectors, animals and humans in this region. *Rhipicephalus pulchellus* is the most predominant tick species in arid and semi-arid regions [44] and the most abundant in cattle of Borana pastoral areas [45]. This tick species has a three-host life cycle and has been shown to infest domestic and large wildlife hosts such as cattle and zebra [44] and therefore there is a possibility that it may serve as a potential reservoir and vector of WNV and other arboviruses.

Arbovirus surveillance studies conducted in Kenya have shown that WNV is transmitted mainly by mosquitoes belonging to the genus *Culex* [30,33]. Therefore, the discovery of WNV in *R. pulchellus* sampled from cattle in Ijara District suggest its potential as a vector for longer distance transfer. This study constitutes the first WNV detection in *R. pulchellus* ticks in Kenya. Vector competence studies performed on *Hyalomma marginatum* in Portugal highlight the role of ticks in the natural transmission of WNV [46]. *Ornithodoros moubata* have also been found to serve as potential reservoirs and vectors of WNV [15].

Phylogenetic analysis of the 27 whole genome sequences including tick-borne WNV isolate obtained from Ijara district in Kenya showed that the Kenyan tick-borne WNV strain belongs to Lineage 1. Other African sequences, mostly those isolated from southern Africa, segregated to form a large cluster consisting of WNV Lineage 2 viruses. Despite being isolated from ticks, WNV strains isolated from mosquitoes and ticks in Kenya formed distinct clade in the time scaled phylogeny (BEAST MCC tree) pointing to cross-species transmission of this virus in Kenya.

Further phylogenetic evolutionary analysis shows that the WNV strain (KC243146.1) isolated from Kenya is evolutionarily related as it clusters together in Bayesian phylogenetic reconstruction. Further observations made from this analysis point to the Japanese encephalitis virus isolate as the most common recent ancestor of the WNV Lineage 1 viruses with the divergence dating back approximately 200 years. Full genome analysis also suggested that the Lineage 1 strains used in this study from Kenya, North Africa and Europe diverged approximately 20 years ago which does not suggest that the Kenyan strain (KC243146.1) was involved in recent introduction. The closest ancestor to the European strains was from Morocco which diverged nine years ago from strains of France and Italy. This does emphasize a possible role of inter- and cross-continental spread for WNV strains. Transfer of ticks through migratory birds remains a potential mechanism to achieve this. Lineage 2 strains diverged more than 200 years ago from Lineage 1. The South African strains diverged 23 years ago from those in Europe. However the Greek strain diverged only nine years ago from those in Hungary; this may suggest that the Central European strains were the origin of this outbreak rather than recent introduction from Africa.

WNV Lineage 1 strains have a widespread distribution in Africa, Europe, the Middle East, America, Australia and India. It is possible that WNV Lineage 1 could have been exported from Africa to Europe in particular, by migratory birds that might have had ticks attached to them though evidence of *R. pulchellus* infesting birds is lacking. The phylogenetic analysis suggests that Morocco rather than Kenya was the source of recent introduction to Europe. Both highly and less neuroinvasive strains have been shown to exist in both Lineage 1 and 2 [7]. Experimental or clinical data on the neurovirulence of Lineage 1 strains from Kenya are not yet available and investigation of WNV as a cause of neurological disease in humans and animals in Kenya is warranted.

Neurovirulence is associated with specific genotypes and not related to lineage, source of isolate, geographic distribution, passage level, or year of isolation [47-49]. Selection analyses carried out in the current study revealed differential selection pressures operating at different gene regions along the WNV genome. In this analysis it was observed that non-synonymous amino acid substitutions are more prevalent in 5′ structural genes region and less prevalent in non-structural gene regions towards the 3′ prime end. Of special interest is the observation that limited or no diversifying selection pressures seemed to be operating on the polymerase gene. This may be attributed to its strategic function in the replication of the virus genome hence being kept under negative selection pressure as seen in Figure 3 (highlighted in blue). The amino acid changes show the same trend in the viruses belonging to similar lineages further underscoring the importance of these genes in the maintenance of progeny, i.e. conferring viral fitness to the various strains. The Kenyan WNV isolate (KC243146.1) from ticks shows amino acid substitutions commonly observed in other strains belonging to Lineage 1 isolated from ticks and mosquitoes from different locations worldwide. These results resonate well with previous studies that have attempted to elucidate genetic markers for the virulence and pathogenicity of different lineages of the WNV strains. According to a study conducted by Beasley [50], enhanced virulence of North American WNV strains compared with other Lineage 1 strains of the Old World was linked to the envelope protein glycosylation. WNV Lineage 2 strains isolated from patients in South Africa all had these envelope protein glycosylation sites which have been associated with increased virulence [51].

The isolation of WNV from *R. pulchellus* species sampled from cattle in Ijara District might imply that either this tick species may have acquired the virus after ingesting a viraemic blood meal from the infected animal or the virus was present in the tick which may imply its potential as vector, reservoir and disseminator of WNV. In general, birds are known as reservoirs of WNV while most mammals have relatively low viremia and act as dead-end hosts [3]. The role of cattle and warthogs as reservoirs for WNV is not known. Although thought to be unlikely, this could be the source of infection to the tick. Surveillance for arboviruses such as WNV should be conducted and appropriate prevention and control strategies be put in place so as to be able to manage arboviruses early enough before they cause outbreaks [52].

## Conclusions

The identification of WNV Lineage 1 in Ijara District further elucidates the geographic range of Lineage 1 and 2 strains in Africa. Lineage 2 strains have been described in Tanzania and are almost exclusively found in South Africa [2] while Lineage 1 strains are the major lineage reported in North Africa, Eurasia, India, the Middle East and the Americas. The close proximity of Europe to Africa may be the reason that both lineages have emerged in Europe although the exact origin of importation of Lineage 2 strains to Europe is not known. Tick vectors pose a feasible transmission/dissemination mechanism for preserving WNV over long distances and extended time if considering the relatively short viremic period in most animals. The Kenyan strain identified here was not close to other published WNV strains if considering the divergence time from known strains. This suggests that a further uncharacterized source of WNV strains exist in livestock and ticks in Africa.

## Additional file

Additional file 1: Table S1. Amino acid substitutions of the WNV polyprotein gene that corresponds to the structural (C, E and M) and non-structural (1, 2A, 2B, 3, 4A, 4B and 5) proteins.
