# Supplementary material for: Whole genome phylogenetic investigation of a West Nile virus strain isolated from a tick sampled from livestock in north eastern Kenya
Source: Parasit Vectors. 2014 Nov 28;7:542. doi: 10.1186/s13071-014-0542-2 (PMC4255437; doi:10.1186/s13071-014-0542-2)
Supplement: Additional file 1: Table S1. — Amino acid substitutions of the WNV polyprotein gene that corresponds to the structural (C, E and M) and non-structural (1, 2A, 2B, 3, 4A, 4B and 5) proteins. [file 13071_2014_542_MOESM1_ESM.pdf]

**Table 1:** Amino acid substitutions of the WNV polyprotein gene that corresponds to the structural (C, E and M) and non-structural (1, 2A, 2B, 3, 4A, 4B and 5) proteins

| | | | | | | | | | | | | | | | | | | | | | | | | | | | | | | | |
|---|---|---|---|---|---|---|---|---|---|---|---|---|---|---|---|---|---|---|---|---|---|---|---|---|---|---|---|---|---|---|---|
| NY99_USA_2005 | M | S | K | K | P | G | G | P | G | K | S | R | A | V | N | M | L | K | R | G | M | P | R | V | L | S | L | I | G | L | [ 30] |
| ATH002316_Kenya_2012 | . | . | . | . | . | . | . | . | . | . | . | . | . | . | . | . | . | . | . | . | . | . | . | . | . | . | . | . | . | . | [ 30] |
| NY99_USA_2005 | K | R | A | M | L | S | L | I | D | G | K | G | P | I | R | F | V | L | A | L | L | A | F | F | R | F | T | A | I | A | [ 60] |
| ATH002316_Kenya_2012 | . | . | . | . | . | . | . | . | . | . | . | . | . | . | . | . | . | . | . | . | . | . | . | . | . | . | . | . | . | . | [ 60] |
| NY99_USA_2005 | P | T | R | A | V | L | D | R | W | R | G | V | N | K | Q | T | A | M | K | H | L | L | S | F | K | K | E | L | G | T | [ 90] |
| ATH002316_Kenya_2012 | . | . | . | . | . | . | . | . | . | . | . | . | . | . | . | . | . | . | . | . | . | . | . | . | . | . | . | . | . | . | [ 90] |
| NY99_USA_2005 | L | T | S | A | I | N | R | R | S | S | K | Q | K | K | R | G | G | K | T | G | I | A | V | M | I | G | L | I | A | S | [ 120] |
| ATH002316_Kenya_2012 | . | . | . | . | . | . | . | . | . | . | . | . | . | . | . | . | . | N | . | . | . | . | . | . | . | . | . | . | . | . | [ 120] |
| NY99_USA_2005 | V | G | A | V | T | L | S | N | F | Q | G | K | V | M | M | T | V | N | A | T | D | V | T | D | V | I | T | I | P | T | [ 150] |
| ATH002316_Kenya_2012 | . | . | . | . | . | . | . | . | . | . | . | . | . | . | . | . | . | . | . | . | . | . | . | . | . | . | . | . | . | . | [ 150] |
| NY99_USA_2005 | A | A | G | K | N | L | C | I | V | R | A | M | D | V | G | Y | M | C | D | D | T | I | T | Y | E | C | P | V | L | S | [ 180] |
| ATH002316_Kenya_2012 | . | . | . | . | . | . | . | . | . | . | . | . | . | . | . | . | . | . | . | . | . | . | . | . | . | . | . | . | . | . | [ 180] |
| NY99_USA_2005 | A | G | N | D | P | E | D | I | D | C | W | C | T | K | S | A | V | Y | V | R | Y | G | R | C | T | K | T | R | H | S | [ 210] |
| ATH002316_Kenya_2012 | . | . | . | . | . | . | . | . | . | . | . | . | . | . | . | . | . | . | . | . | . | . | . | . | . | . | . | . | . | . | [ 210] |
| NY99_USA_2005 | R | R | S | R | R | S | L | T | V | Q | T | H | G | E | S | T | L | A | N | K | K | G | A | W | M | D | S | T | K | A | [ 240] |
| ATH002316_Kenya_2012 | . | . | . | . | . | . | . | . | . | . | . | . | . | . | . | . | . | . | . | . | . | . | . | . | . | . | . | . | . | . | [ 240] |
| NY99_USA_2005 | T | R | Y | L | V | K | T | E | S | W | I | L | R | N | P | G | Y | A | L | V | A | A | V | I | G | W | M | L | G | S | [ 270] |
| ATH002316_Kenya_2012 | . | . | . | . | . | . | . | . | . | . | . | . | . | . | . | . | . | . | . | . | . | . | . | . | . | . | . | . | . | . | [ 270] |
| NY99_USA_2005 | N | T | M | Q | R | V | V | F | V | V | L | L | L | L | V | A | P | A | Y | S | F | N | C | L | G | M | S | N | R | D | [ 300] |
| ATH002316_Kenya_2012 | . | . | . | . | . | . | . | . | . | . | . | . | . | . | . | . | . | . | . | . | . | . | . | . | . | . | . | . | . | . | [ 300] |
| NY99_USA_2005 | F | L | E | G | V | S | G | A | T | W | V | D | L | V | L | E | G | D | S | C | V | T | I | M | S | K | D | K | P | T | [ 330] |

| | | | | | | | | | | | | | | | | | | | | | | | | | | | | | | | |
|---|---|---|---|---|---|---|---|---|---|---|---|---|---|---|---|---|---|---|---|---|---|---|---|---|---|---|---|---|---|---|---|
| ATH002316_Kenya_2012 | . | . | . | . | . | . | . | . | . | . | . | . | . | . | . | . | . | . | . | . | . | . | . | . | . | . | . | . | . | . | [ 330] |
| NY99_USA_2005 | I | D | V | K | M | M | N | M | E | A | A | N | L | A | E | V | R | S | Y | C | Y | L | A | T | V | S | D | L | S | T | [ 360] |
| ATH002316_Kenya_2012 | . | . | . | . | . | . | . | . | . | . | . | . | . | . | . | . | . | . | . | . | . | . | . | . | . | . | . | . | . | . | [ 360] |
| NY99_USA_2005 | K | A | A | C | P | T | M | G | E | A | H | N | D | K | R | A | D | P | A | F | V | C | R | Q | G | V | V | D | R | G | [ 390] |
| ATH002316_Kenya_2012 | . | . | . | . | . | . | . | . | . | . | . | . | . | . | . | . | . | . | . | . | . | . | . | . | . | . | . | . | . | . | [ 390] |
| NY99_USA_2005 | W | G | N | G | C | G | L | F | G | K | G | S | I | D | T | C | A | K | F | A | C | S | T | K | A | I | G | R | T | I | [ 420] |
| ATH002316_Kenya_2012 | . | . | . | . | . | . | . | . | . | . | . | . | . | . | . | . | . | . | . | . | . | . | . | . | . | T | . | . | . | . | [ 420] |
| NY99_USA_2005 | L | K | E | N | I | K | Y | E | V | A | I | F | V | H | G | P | T | T | V | E | S | H | G | N | Y | S | T | Q | V | G | [ 450] |
| ATH002316_Kenya_2012 | . | . | . | . | . | . | . | . | . | . | . | . | . | . | . | . | . | . | . | . | . | . | . | . | . | . | . | . | I | . | [ 450] |
| NY99_USA_2005 | A | T | Q | A | G | R | L | S | I | T | P | A | A | P | S | Y | T | L | K | L | G | E | Y | G | E | V | T | V | D | C | [ 480] |
| ATH002316_Kenya_2012 | . | . | . | . | . | . | F | . | . | . | . | . | . | . | . | . | . | . | . | . | . | . | . | . | . | . | . | . | . | . | [ 480] |
| NY99_USA_2005 | E | P | R | S | G | I | D | T | N | A | Y | Y | V | M | T | V | G | T | K | T | F | L | V | H | R | E | W | F | M | D | [ 510] |
| ATH002316_Kenya_2012 | . | . | . | . | . | . | . | . | . | . | . | . | . | . | . | . | . | . | . | . | . | . | . | . | . | . | . | . | . | . | [ 510] |
| NY99_USA_2005 | L | N | L | P | W | S | S | A | G | S | T | V | W | R | N | R | E | T | L | M | E | F | E | E | P | H | A | T | K | Q | [ 540] |
| ATH002316_Kenya_2012 | . | . | . | . | . | . | . | . | . | . | . | . | . | . | . | . | . | . | . | . | . | . | . | . | . | . | . | . | . | . | [ 540] |
| NY99_USA_2005 | S | V | I | A | L | G | S | Q | E | G | A | L | H | Q | A | L | A | G | A | I | P | V | E | F | S | S | N | T | V | K | [ 570] |
| ATH002316_Kenya_2012 | . | . | . | . | . | . | . | . | G | . | R | S | C | I | K | . | W | L | E | P | S | L | W | I | . | . | . | . | . | . | [ 570] |
| NY99_USA_2005 | L | T | S | G | H | L | K | C | R | V | K | M | E | K | L | Q | L | K | G | T | T | Y | G | V | C | S | K | A | F | K | [ 600] |
| ATH002316_Kenya_2012 | . | . | . | . | . | . | . | . | . | . | . | . | . | . | . | . | . | . | . | . | . | . | . | . | . | . | . | . | . | . | [ 600] |
| NY99_USA_2005 | F | L | G | T | P | A | D | T | G | H | G | T | V | V | L | E | L | Q | Y | T | G | T | D | G | P | C | K | V | P | I | [ 630] |
| ATH002316_Kenya_2012 | . | . | . | . | . | . | V | . | . | . | . | . | . | . | . | . | . | . | . | . | . | . | . | . | . | . | . | . | . | . | [ 630] |
| NY99_USA_2005 | S | S | V | A | S | L | N | D | L | T | P | V | G | R | L | V | T | V | N | P | F | V | S | V | A | T | A | N | A | K | [ 660] |
| ATH002316_Kenya_2012 | . | . | . | . | . | . | . | . | . | . | . | . | . | . | . | . | . | . | . | . | . | . | . | . | S | . | . | . | . | . | [ 660] |
| NY99_USA_2005 | V | L | I | E | L | E | P | P | F | G | D | S | Y | I | V | V | G | R | G | E | Q | Q | I | N | H | H | W | H | K | S | [ 690] |

| | | | | | | | | | | | | | | | | | | | | | | | | | | | | | | | |
|---|---|---|---|---|---|---|---|---|---|---|---|---|---|---|---|---|---|---|---|---|---|---|---|---|---|---|---|---|---|---|---|
| ATH002316_Kenya_2012 | . | . | . | . | . | . | . | . | . | . | . | . | . | . | . | . | . | . | . | . | . | . | . | . | . | . | . | . | . | . | [ 690] |
| NY99_USA_2005 | G | S | S | I | G | K | A | F | T | T | T | L | K | G | A | Q | R | L | A | A | L | G | D | T | A | W | D | F | G | S | [ 720] |
| ATH002316_Kenya_2012 | . | . | . | . | . | . | . | . | . | . | . | . | . | . | . | . | . | . | . | . | . | . | . | . | . | . | . | . | . | . | [ 720] |
| NY99_USA_2005 | V | G | G | V | F | T | S | V | G | K | A | V | H | Q | V | F | G | G | A | F | R | S | L | F | G | G | M | S | W | I | [ 750] |
| ATH002316_Kenya_2012 | . | . | . | . | . | . | . | . | . | . | . | . | . | . | . | . | . | . | . | . | . | . | . | . | . | . | . | . | . | . | [ 750] |
| NY99_USA_2005 | T | Q | G | L | L | G | A | L | L | L | W | M | G | I | N | A | R | D | R | S | I | A | L | T | F | L | A | V | G | G | [ 780] |
| ATH002316_Kenya_2012 | . | . | . | . | . | . | . | . | . | . | . | . | . | . | . | . | . | . | . | . | . | . | . | . | . | . | . | . | . | . | [ 780] |
| NY99_USA_2005 | V | L | L | F | L | S | V | N | V | H | A | D | T | G | C | A | I | D | I | S | R | Q | E | L | R | C | G | S | G | V | [ 810] |
| ATH002316_Kenya_2012 | . | . | . | . | . | . | . | . | . | . | . | . | . | . | . | . | . | . | . | . | . | . | . | . | . | . | . | . | . | . | [ 810] |
| NY99_USA_2005 | F | I | H | N | D | V | E | A | W | M | D | R | Y | K | Y | Y | P | E | T | P | Q | G | L | A | K | I | I | Q | K | A | [ 840] |
| ATH002316_Kenya_2012 | . | . | . | . | . | . | . | . | . | . | . | . | . | . | . | . | . | . | . | . | . | . | . | . | . | . | . | . | . | . | [ 840] |
| NY99_USA_2005 | H | K | E | G | V | C | G | L | R | S | V | S | R | L | E | H | Q | M | W | E | A | V | K | D | E | L | N | T | L | L | [ 870] |
| ATH002316_Kenya_2012 | . | . | . | . | . | . | . | . | . | . | . | . | . | . | . | . | . | . | . | . | S | . | . | . | . | . | . | . | . | . | [ 870] |
| NY99_USA_2005 | K | E | N | G | V | D | L | S | V | V | V | E | K | Q | E | G | M | Y | K | S | A | P | K | R | L | T | A | T | T | E | [ 900] |
| ATH002316_Kenya_2012 | . | . | . | . | . | . | . | . | . | . | . | . | . | . | . | . | . | . | . | . | . | . | . | . | . | . | . | . | . | . | [ 900] |
| NY99_USA_2005 | K | L | E | I | G | W | K | A | W | G | K | S | I | L | F | A | P | E | L | A | N | N | T | F | V | V | D | G | P | E | [ 930] |
| ATH002316_Kenya_2012 | . | . | . | . | . | . | . | . | . | . | . | . | . | . | . | . | . | . | . | . | . | . | . | . | . | . | . | . | . | . | [ 930] |
| NY99_USA_2005 | T | K | E | C | P | T | Q | N | R | A | W | N | S | L | E | V | E | D | F | G | F | G | L | T | S | T | R | M | F | L | [ 960] |
| ATH002316_Kenya_2012 | . | . | . | . | . | . | . | . | . | . | . | . | . | . | . | . | . | . | . | . | . | . | . | . | . | . | . | . | . | . | [ 960] |
| NY99_USA_2005 | K | V | R | E | S | N | T | T | E | C | D | S | K | I | I | G | T | A | V | K | N | N | L | A | I | H | S | D | L | S | [ 990] |
| ATH002316_Kenya_2012 | . | . | . | . | . | . | . | . | . | . | . | . | . | . | . | . | . | . | . | . | . | . | . | . | . | . | . | . | . | . | [ 990] |
| NY99_USA_2005 | Y | W | I | E | S | R | L | N | D | T | W | K | L | E | R | A | V | L | G | E | V | K | S | C | T | W | P | E | T | H | [1020] |
| ATH002316_Kenya_2012 | . | . | . | . | . | . | . | . | . | . | . | . | . | . | . | . | . | . | . | . | . | . | . | . | . | . | . | . | . | . | [1020] |
| NY99_USA_2005 | T | L | W | G | D | G | I | L | E | S | D | L | I | I | P | V | T | L | A | G | P | R | S | N | H | N | R | R | P | G | [1050] |

| | | | | | | | | | | | | | | | | | | | | | | | | | | | | | | | |
|---|---|---|---|---|---|---|---|---|---|---|---|---|---|---|---|---|---|---|---|---|---|---|---|---|---|---|---|---|---|---|---|
| ATH002316_Kenya_2012 | . | . | . | . | . | . | . | . | . | . | . | . | . | . | . | . | . | . | . | . | . | . | . | . | . | . | . | . | . | . | [1050] |
| NY99_USA_2005 | Y | K | T | Q | N | Q | G | P | W | D | E | G | R | V | E | I | D | F | D | Y | C | P | G | T | T | V | T | L | S | E | [1080] |
| ATH002316_Kenya_2012 | . | . | . | . | . | . | . | . | . | . | . | . | . | . | . | . | . | . | . | . | . | . | . | . | . | . | . | . | . | . | [1080] |
| NY99_USA_2005 | S | C | G | H | R | G | P | A | T | R | T | T | T | E | S | G | K | L | I | T | D | W | C | C | R | S | C | T | L | P | [1110] |
| ATH002316_Kenya_2012 | . | . | . | . | . | . | . | . | . | . | . | . | . | . | . | . | . | . | . | A | . | . | . | . | . | . | . | . | . | . | [1110] |
| NY99_USA_2005 | P | L | R | Y | Q | T | D | S | G | C | W | Y | G | M | E | I | R | P | Q | R | H | D | E | K | T | L | V | Q | S | Q | [1140] |
| ATH002316_Kenya_2012 | . | . | . | . | . | . | . | . | . | . | . | . | . | . | . | . | . | . | . | . | . | . | . | . | . | . | . | . | . | . | [1140] |
| NY99_USA_2005 | V | N | A | Y | N | A | D | M | I | D | P | F | Q | L | G | L | L | V | V | F | L | A | T | Q | E | V | L | R | K | R | [1170] |
| ATH002316_Kenya_2012 | . | . | . | . | . | . | . | . | . | . | . | . | . | . | . | . | . | . | . | . | . | . | . | . | . | . | . | . | . | . | [1170] |
| NY99_USA_2005 | W | T | A | K | I | S | M | P | A | I | L | I | A | L | L | V | L | V | F | G | G | I | T | Y | T | D | V | L | R | Y | [1200] |
| ATH002316_Kenya_2012 | . | . | . | . | . | . | . | . | . | . | . | . | . | . | . | . | . | . | . | . | . | . | . | . | . | . | . | . | . | . | [1200] |
| NY99_USA_2005 | V | I | L | V | G | A | A | F | A | E | S | N | S | G | G | D | V | V | H | L | A | L | M | A | T | F | K | I | Q | P | [1230] |
| ATH002316_Kenya_2012 | . | . | . | . | . | . | . | . | . | . | . | . | . | . | . | . | . | . | . | . | . | . | . | . | . | . | . | . | . | . | [1230] |
| NY99_USA_2005 | V | F | M | V | A | S | F | L | K | A | R | W | T | N | Q | E | N | I | L | L | M | L | A | A | V | F | F | Q | M | A | [1260] |
| ATH002316_Kenya_2012 | . | . | . | . | . | . | . | . | . | . | . | . | . | . | . | . | . | . | . | . | . | M | . | . | . | . | . | . | . | . | [1260] |
| NY99_USA_2005 | Y | H | D | A | R | Q | I | L | L | W | E | I | P | D | V | L | N | S | L | A | V | A | W | M | I | L | R | A | I | T | [1290] |
| ATH002316_Kenya_2012 | . | . | . | . | . | . | . | . | . | . | . | . | . | . | . | . | . | . | . | . | . | . | . | . | . | . | . | . | . | . | [1290] |
| NY99_USA_2005 | F | T | T | T | S | N | V | V | V | P | L | L | A | L | L | T | P | G | L | R | C | L | N | L | D | V | Y | R | I | L | [1320] |
| ATH002316_Kenya_2012 | . | . | . | . | . | . | . | . | . | . | . | . | . | . | . | . | . | . | . | . | . | . | . | . | . | . | . | . | . | . | [1320] |
| NY99_USA_2005 | L | L | M | V | G | I | G | S | L | I | R | E | K | R | S | A | A | A | K | K | K | G | A | S | L | L | C | L | A | L | [1350] |
| ATH002316_Kenya_2012 | . | . | . | . | . | . | . | . | . | . | . | . | . | . | . | . | . | . | . | . | . | . | . | . | . | . | . | . | . | . | [1350] |
| NY99_USA_2005 | A | S | T | G | L | F | N | P | M | I | L | A | A | G | L | I | A | C | D | P | N | R | K | R | G | W | P | A | T | E | [1380] |
| ATH002316_Kenya_2012 | . | . | . | . | . | . | . | . | . | . | . | . | . | . | . | . | . | . | . | . | . | . | . | . | . | . | . | . | . | . | [1380] |
| NY99_USA_2005 | V | M | T | A | V | G | L | M | F | A | I | V | G | G | L | A | E | L | D | I | D | S | M | A | I | P | M | T | I | A | [1410] |

| | | | | | | | | | | | | | | | | | | | | | | | | | | | | | | | |
|---|---|---|---|---|---|---|---|---|---|---|---|---|---|---|---|---|---|---|---|---|---|---|---|---|---|---|---|---|---|---|---|
| ATH002316_Kenya_2012 | . | . | . | . | . | . | . | . | . | . | . | . | . | . | . | . | . | . | . | . | . | . | . | . | . | . | . | . | . | . | [1410] |
| NY99_USA_2005 | G | L | M | F | A | A | F | V | I | S | G | K | S | T | D | M | W | I | E | R | T | A | D | I | S | W | E | S | D | A | [1440] |
| ATH002316_Kenya_2012 | . | . | . | . | . | . | . | . | . | . | . | . | . | . | . | . | . | . | . | . | . | . | . | . | . | . | . | . | . | . | [1440] |
| NY99_USA_2005 | E | I | T | G | S | S | E | R | V | D | V | R | L | D | D | D | G | N | F | Q | L | M | N | D | P | G | A | P | W | K | [1470] |
| ATH002316_Kenya_2012 | . | . | . | . | . | . | . | . | . | . | . | . | . | . | . | . | . | . | . | . | . | . | . | . | . | . | . | . | . | . | [1470] |
| NY99_USA_2005 | I | W | M | L | R | M | V | C | L | A | I | S | A | Y | T | P | W | A | I | L | P | S | V | V | G | F | W | I | T | L | [1500] |
| ATH002316_Kenya_2012 | . | . | . | . | . | . | A | . | . | . | . | . | . | . | . | . | . | . | . | . | . | . | . | . | . | . | . | . | . | . | [1500] |
| NY99_USA_2005 | Q | Y | T | K | R | G | G | V | L | W | D | T | P | S | P | K | E | Y | K | K | G | D | T | T | T | G | V | Y | R | I | [1530] |
| ATH002316_Kenya_2012 | . | . | . | . | . | . | . | . | . | . | . | . | . | . | . | . | . | . | . | . | . | . | . | . | . | . | . | . | . | . | [1530] |
| NY99_USA_2005 | M | T | R | G | L | L | G | S | Y | Q | A | G | A | G | V | M | V | E | G | V | F | H | T | L | W | H | T | T | K | G | [1560] |
| ATH002316_Kenya_2012 | . | . | . | . | . | . | . | . | . | . | . | . | . | . | . | . | . | . | . | . | . | . | . | . | . | . | . | . | . | . | [1560] |
| NY99_USA_2005 | A | A | L | M | S | G | E | G | R | L | D | P | Y | W | G | S | V | K | E | D | R | L | C | Y | G | G | P | W | K | L | [1590] |
| ATH002316_Kenya_2012 | . | . | . | . | . | . | . | . | . | . | . | . | . | . | . | . | . | . | . | . | . | . | . | . | . | . | . | . | . | . | [1590] |
| NY99_USA_2005 | Q | H | K | W | N | G | Q | D | E | V | Q | M | I | V | V | E | P | G | K | N | V | K | N | V | Q | T | K | P | G | V | [1620] |
| ATH002316_Kenya_2012 | . | . | . | . | . | . | . | . | . | . | . | . | . | . | . | . | . | . | . | . | . | . | . | . | . | . | . | . | . | . | [1620] |
| NY99_USA_2005 | F | K | T | P | E | G | E | I | G | A | V | T | L | D | F | P | T | G | T | S | G | S | P | I | V | D | K | N | G | D | [1650] |
| ATH002316_Kenya_2012 | . | . | . | . | . | . | . | . | . | . | . | . | . | . | . | . | . | . | . | . | . | . | . | . | . | . | . | . | . | . | [1650] |
| NY99_USA_2005 | V | I | G | L | Y | G | N | G | V | I | M | P | N | G | S | Y | I | S | A | I | V | Q | G | E | R | M | D | E | P | I | [1680] |
| ATH002316_Kenya_2012 | . | . | . | . | . | . | . | . | . | . | . | . | . | . | . | . | . | . | . | . | . | . | . | . | . | . | . | . | . | . | [1680] |
| NY99_USA_2005 | P | A | G | F | E | P | E | M | L | R | K | K | Q | I | T | V | L | D | L | H | P | G | A | G | K | T | R | R | I | L | [1710] |
| ATH002316_Kenya_2012 | . | . | . | . | . | . | . | . | . | . | . | . | . | . | . | . | . | . | . | . | . | . | . | . | . | . | . | . | . | . | [1710] |
| NY99_USA_2005 | P | Q | I | I | K | E | A | I | N | R | R | L | R | T | A | V | L | A | P | T | R | V | V | A | A | E | M | A | E | A | [1740] |
| ATH002316_Kenya_2012 | . | . | . | . | . | . | . | . | . | . | . | . | . | . | . | . | . | . | . | . | . | . | . | . | . | . | . | . | . | . | [1740] |
| NY99_USA_2005 | L | R | G | L | P | I | R | Y | Q | T | S | A | V | P | R | E | H | N | G | N | E | I | V | D | V | M | C | H | A | T | [1770] |

| | | | | | | | | | | | | | | | | | | | | | | | | | | | | | | | |
|---|---|---|---|---|---|---|---|---|---|---|---|---|---|---|---|---|---|---|---|---|---|---|---|---|---|---|---|---|---|---|---|
| ATH002316_Kenya_2012 | . | . | . | . | . | . | . | . | . | . | . | . | . | T | . | . | . | . | . | . | . | . | . | . | . | . | . | . | . | . | [1770] |
| NY99_USA_2005 | L | T | H | R | L | M | S | P | H | R | V | P | N | Y | N | L | F | V | M | D | E | A | H | F | T | D | P | A | S | I | [1800] |
| ATH002316_Kenya_2012 | . | . | . | . | . | . | . | . | . | . | . | . | . | . | . | . | . | . | . | . | . | . | . | . | . | . | . | . | . | . | [1800] |
| NY99_USA_2005 | A | A | R | G | Y | I | S | T | K | V | E | L | G | E | A | A | A | I | F | M | T | A | T | P | P | G | T | S | D | P | [1830] |
| ATH002316_Kenya_2012 | . | . | . | . | . | . | . | . | . | . | . | . | . | . | . | . | . | . | . | . | . | . | . | . | . | . | . | . | . | . | [1830] |
| NY99_USA_2005 | F | P | E | S | N | S | P | I | S | D | L | Q | T | E | I | P | D | R | A | W | N | S | G | Y | E | W | I | T | E | Y | [1860] |
| ATH002316_Kenya_2012 | . | . | . | . | . | . | . | . | . | . | . | . | . | . | . | . | . | . | . | . | . | . | . | . | . | . | . | . | . | . | [1860] |
| NY99_USA_2005 | T | G | K | T | V | W | F | V | P | S | V | K | M | G | N | E | I | A | L | C | L | Q | R | A | G | K | K | V | V | Q | [1890] |
| ATH002316_Kenya_2012 | I | . | . | . | . | . | . | . | . | . | . | . | . | . | . | . | . | . | . | . | . | . | . | . | . | . | . | . | . | . | [1890] |
| NY99_USA_2005 | L | N | R | K | S | Y | E | T | E | Y | P | K | C | K | N | D | D | W | D | F | V | I | T | T | D | I | S | E | M | G | [1920] |
| ATH002316_Kenya_2012 | . | . | . | . | . | . | . | . | . | . | . | . | . | . | . | . | . | . | . | . | . | . | . | . | . | . | . | . | . | . | [1920] |
| NY99_USA_2005 | A | N | F | K | A | S | R | V | I | D | S | R | K | S | V | K | P | T | I | I | T | E | G | E | G | R | V | I | L | G | [1950] |
| ATH002316_Kenya_2012 | . | . | . | . | . | . | . | . | . | . | . | . | . | . | . | . | . | . | . | . | . | . | . | . | . | . | . | . | . | . | [1950] |
| NY99_USA_2005 | E | P | S | A | V | T | A | A | S | A | A | Q | R | R | G | R | I | G | R | N | P | S | Q | V | G | D | E | Y | C | Y | [1980] |
| ATH002316_Kenya_2012 | . | . | . | . | . | . | . | . | . | . | . | . | . | . | . | . | . | . | . | . | . | . | . | . | . | . | . | . | . | . | [1980] |
| NY99_USA_2005 | G | G | H | T | N | E | D | D | S | N | F | A | H | W | T | E | A | R | I | M | L | D | N | I | N | M | P | N | G | L | [2010] |
| ATH002316_Kenya_2012 | . | . | . | . | . | . | . | . | . | . | . | . | . | . | . | . | . | . | . | . | . | . | . | . | . | . | . | . | . | . | [2010] |
| NY99_USA_2005 | I | A | Q | F | Y | Q | P | E | R | E | K | V | Y | T | M | D | G | E | Y | R | L | R | G | E | E | R | K | N | F | L | [2040] |
| ATH002316_Kenya_2012 | . | . | . | . | . | . | . | . | . | . | . | . | . | . | . | . | . | . | . | . | . | . | . | . | . | . | . | . | . | . | [2040] |
| NY99_USA_2005 | E | L | L | R | T | A | D | L | P | V | W | L | A | Y | K | V | A | A | A | G | V | S | Y | H | D | R | R | W | C | F | [2070] |
| ATH002316_Kenya_2012 | . | . | . | . | . | . | . | . | . | . | . | . | . | . | . | . | . | . | . | . | . | . | . | . | . | . | . | . | . | . | [2070] |
| NY99_USA_2005 | D | G | P | R | T | N | T | I | L | E | D | N | N | E | V | E | V | I | T | K | L | G | E | R | K | I | L | R | P | R | [2100] |
| ATH002316_Kenya_2012 | . | . | . | . | . | . | . | . | . | . | . | . | . | . | . | . | . | . | . | . | . | . | . | . | . | . | . | . | . | . | [2100] |
| NY99_USA_2005 | W | I | D | A | R | V | Y | S | D | H | Q | A | L | K | A | F | K | D | F | A | S | G | K | R | S | Q | I | G | L | I | [2130] |

| | | | | | | | | | | | | | | | | | | | | | | | | | | | | | | | |
|---|---|---|---|---|---|---|---|---|---|---|---|---|---|---|---|---|---|---|---|---|---|---|---|---|---|---|---|---|---|---|---|
| ATH002316_Kenya_2012 | . | . | . | . | . | . | . | . | . | . | . | . | . | . | . | . | . | . | . | . | . | . | . | . | . | . | . | . | . | . | [2130] |
| NY99_USA_2005 | E | V | L | G | K | M | P | E | H | F | M | G | K | T | W | E | A | L | D | T | M | Y | V | V | A | T | A | E | K | G | [2160] |
| ATH002316_Kenya_2012 | . | . | . | . | . | . | . | . | . | . | . | . | . | . | . | . | . | . | . | . | . | . | . | . | . | . | . | . | . | . | [2160] |
| NY99_USA_2005 | G | R | A | H | R | M | A | L | E | E | L | P | D | A | L | Q | T | I | A | L | I | A | L | L | S | V | M | T | M | G | [2190] |
| ATH002316_Kenya_2012 | . | . | . | . | . | . | . | . | . | . | . | . | . | . | . | . | . | . | . | . | . | . | . | . | . | . | . | . | . | . | [2190] |
| NY99_USA_2005 | V | F | F | L | L | M | Q | R | K | G | I | G | K | I | G | L | G | G | A | V | L | G | V | A | T | F | F | C | W | M | [2220] |
| ATH002316_Kenya_2012 | . | . | . | . | . | . | . | . | . | . | . | . | . | . | . | . | . | . | V | . | . | . | . | . | . | . | . | . | . | . | [2220] |
| NY99_USA_2005 | A | E | V | P | G | T | K | I | A | G | M | L | L | L | S | L | L | L | M | I | V | L | I | P | E | P | E | K | Q | R | [2250] |
| ATH002316_Kenya_2012 | . | . | . | . | . | . | . | . | . | . | . | . | . | . | . | . | . | . | . | . | . | . | . | . | . | . | . | . | . | . | [2250] |
| NY99_USA_2005 | S | Q | T | D | N | Q | L | A | V | F | L | I | C | V | M | T | L | V | S | A | V | A | A | N | E | M | G | W | L | D | [2280] |
| ATH002316_Kenya_2012 | . | . | . | . | . | . | . | . | . | . | . | . | . | . | . | . | . | . | . | . | . | . | . | . | . | . | . | . | . | . | [2280] |
| NY99_USA_2005 | K | T | K | S | D | I | S | S | L | F | G | Q | R | I | E | V | K | E | N | F | S | M | G | E | F | L | L | D | L | R | [2310] |
| ATH002316_Kenya_2012 | . | . | . | . | . | . | . | . | . | . | . | . | . | . | . | . | . | . | . | . | . | . | . | . | . | . | . | . | . | . | [2310] |
| NY99_USA_2005 | P | A | T | A | W | S | L | Y | A | V | T | T | A | V | L | T | P | L | L | K | H | L | I | T | S | D | Y | I | N | T | [2340] |
| ATH002316_Kenya_2012 | . | . | . | . | . | . | . | . | . | . | . | . | . | . | . | . | . | . | . | . | . | . | . | . | . | . | . | . | . | . | [2340] |
| NY99_USA_2005 | S | L | T | S | I | N | V | Q | A | S | A | L | F | T | L | A | R | G | F | P | F | V | D | V | G | V | S | A | L | L | [2370] |
| ATH002316_Kenya_2012 | . | . | . | . | . | . | . | . | . | . | . | . | . | . | . | . | . | . | . | . | . | . | . | . | . | . | . | . | . | . | [2370] |
| NY99_USA_2005 | L | A | A | G | C | W | G | Q | V | T | L | T | V | T | V | T | A | A | T | L | L | F | C | H | Y | A | Y | M | V | P | [2400] |
| ATH002316_Kenya_2012 | . | . | . | . | S | . | . | . | . | . | . | . | . | . | . | . | . | . | . | . | . | . | . | . | . | . | . | . | . | . | [2400] |
| NY99_USA_2005 | G | W | Q | A | E | A | M | R | S | A | Q | R | R | T | A | A | G | I | M | K | N | A | V | V | D | G | I | V | A | T | [2430] |
| ATH002316_Kenya_2012 | . | . | . | . | . | . | . | . | . | . | . | . | . | . | . | . | . | . | . | . | . | . | . | . | . | . | . | . | . | . | [2430] |
| NY99_USA_2005 | D | V | P | E | L | E | R | T | T | P | I | M | Q | K | K | V | G | Q | I | M | L | I | L | V | S | L | A | A | V | V | [2460] |
| ATH002316_Kenya_2012 | . | . | . | . | . | . | . | . | . | . | . | . | . | . | . | . | . | . | . | . | . | . | . | . | . | . | . | . | . | . | [2460] |
| NY99_USA_2005 | V | N | P | S | V | K | T | V | R | E | A | G | I | L | I | T | A | A | A | V | T | L | W | E | N | G | A | S | S | V | [2490] |

| | | | | | | | | | | | | | | | | | | | | | | | | | | | | | | | |
|---|---|---|---|---|---|---|---|---|---|---|---|---|---|---|---|---|---|---|---|---|---|---|---|---|---|---|---|---|---|---|---|
| ATH002316_Kenya_2012 | . | . | . | . | . | . | . | . | . | . | . | . | . | . | . | . | . | . | . | . | . | . | . | . | . | . | . | . | . | . | [2490] |
| NY99_USA_2005 | W | N | A | T | T | A | I | G | L | C | H | I | M | R | G | G | W | L | S | C | L | S | I | T | W | T | L | I | K | N | [2520] |
| ATH002316_Kenya_2012 | . | . | . | . | . | . | . | . | . | . | . | . | . | . | . | . | . | . | . | . | . | . | . | . | . | . | . | . | . | . | [2520] |
| NY99_USA_2005 | M | E | K | P | G | L | K | R | G | G | A | K | G | R | T | L | G | E | V | W | K | E | R | L | N | Q | M | T | K | E | [2550] |
| ATH002316_Kenya_2012 | . | D | . | . | . | . | . | . | . | . | . | . | . | . | . | . | . | . | . | . | . | . | . | . | . | . | . | . | . | . | [2550] |
| NY99_USA_2005 | E | F | T | R | Y | R | K | E | A | I | I | E | V | D | R | S | A | A | K | H | A | R | K | E | G | N | V | T | G | G | [2580] |
| ATH002316_Kenya_2012 | . | . | . | . | . | . | . | . | . | . | . | . | . | . | . | . | . | . | . | . | . | . | . | . | . | . | . | . | . | . | [2580] |
| NY99_USA_2005 | H | P | V | S | R | G | T | A | K | L | R | W | L | V | E | R | R | F | L | E | P | V | G | K | V | I | D | L | G | C | [2610] |
| ATH002316_Kenya_2012 | . | . | . | . | . | . | . | . | . | . | . | . | . | . | . | . | . | . | . | . | . | . | . | . | . | . | . | . | . | . | [2610] |
| NY99_USA_2005 | G | R | G | G | W | C | Y | Y | M | A | T | Q | K | R | V | Q | E | V | R | G | Y | T | K | G | G | P | G | H | E | E | [2640] |
| ATH002316_Kenya_2012 | . | . | . | . | . | . | . | . | . | . | . | . | . | . | . | . | . | . | . | . | . | . | . | . | . | . | . | . | . | . | [2640] |
| NY99_USA_2005 | P | Q | L | V | Q | S | Y | G | W | N | I | V | T | M | K | S | G | V | D | V | F | Y | R | P | S | E | C | C | D | T | [2670] |
| ATH002316_Kenya_2012 | . | . | . | . | . | . | . | . | . | . | . | . | . | . | . | . | . | . | . | . | . | . | . | . | . | . | . | . | . | . | [2670] |
| NY99_USA_2005 | L | L | C | D | I | G | E | S | S | S | S | A | E | V | E | E | H | R | T | I | R | V | L | E | M | V | E | D | W | L | [2700] |
| ATH002316_Kenya_2012 | . | . | . | . | . | . | . | . | . | . | . | . | . | . | . | . | . | . | . | . | . | . | . | . | . | . | . | . | . | . | [2700] |
| NY99_USA_2005 | H | R | G | P | R | E | F | C | V | K | V | L | C | P | Y | M | P | K | V | I | E | K | M | E | L | L | Q | R | R | Y | [2730] |
| ATH002316_Kenya_2012 | . | . | . | . | . | . | . | . | . | . | . | . | . | . | . | . | . | . | . | . | . | . | . | . | . | . | . | . | . | . | [2730] |
| NY99_USA_2005 | G | G | G | L | V | R | N | P | L | S | R | N | S | T | H | E | M | Y | W | V | S | R | A | S | G | N | V | V | H | S | [2760] |
| ATH002316_Kenya_2012 | . | . | . | . | . | . | . | . | . | . | . | . | . | . | . | . | . | . | . | . | . | . | . | . | . | . | . | . | . | . | [2760] |
| NY99_USA_2005 | V | N | M | T | S | Q | V | L | L | G | R | M | E | K | R | T | W | K | G | P | Q | Y | E | E | D | V | N | L | G | S | [2790] |
| ATH002316_Kenya_2012 | . | . | . | . | . | . | . | . | . | . | . | . | . | . | . | . | . | . | . | . | . | . | . | . | . | . | . | . | . | . | [2790] |
| NY99_USA_2005 | G | T | R | A | V | G | K | P | L | L | N | S | D | T | S | K | I | K | N | R | I | E | R | L | R | R | E | Y | S | S | [2820] |
| ATH002316_Kenya_2012 | . | . | . | . | . | . | . | . | . | . | . | . | . | . | . | . | . | . | . | . | . | . | . | . | . | . | . | . | . | . | [2820] |
| NY99_USA_2005 | T | W | H | H | D | E | N | H | P | Y | R | T | W | N | Y | H | G | S | Y | D | V | K | P | T | G | S | A | S | S | L | [2850] |

| | | | | | | | | | | | | | | | | | | | | | | | | | | | | | | | |
|---|---|---|---|---|---|---|---|---|---|---|---|---|---|---|---|---|---|---|---|---|---|---|---|---|---|---|---|---|---|---|---|
| ATH002316_Kenya_2012 | . | . | . | . | . | . | . | . | . | . | . | . | . | . | . | . | . | . | . | . | . | . | . | . | . | . | . | . | . | . | [2850] |
| NY99_USA_2005 | V | N | G | V | V | R | L | L | S | K | P | W | D | T | I | T | N | V | T | T | M | A | M | T | D | T | T | P | F | G | [2880] |
| ATH002316_Kenya_2012 | . | . | . | . | . | . | . | . | . | . | . | . | . | . | . | . | . | . | . | . | . | . | . | . | . | . | . | . | . | . | [2880] |
| NY99_USA_2005 | Q | Q | R | V | F | K | E | K | V | D | T | K | A | P | E | P | P | E | G | V | K | Y | V | L | N | E | T | T | N | W | [2910] |
| ATH002316_Kenya_2012 | . | . | . | . | . | . | . | . | . | . | . | . | . | . | . | . | . | . | . | . | . | . | . | . | . | . | . | . | . | . | [2910] |
| NY99_USA_2005 | L | W | A | F | L | A | R | E | K | R | P | R | M | C | S | R | E | E | F | I | R | K | V | N | S | N | A | A | L | G | [2940] |
| ATH002316_Kenya_2012 | . | . | . | . | . | . | . | . | . | . | . | . | . | . | . | . | . | . | . | . | . | . | . | . | . | . | . | . | . | . | [2940] |
| NY99_USA_2005 | A | M | F | E | E | Q | N | Q | W | R | S | A | R | E | A | V | E | D | P | K | F | W | E | M | V | D | E | E | R | E | [2970] |
| ATH002316_Kenya_2012 | . | . | . | . | . | . | . | . | . | . | . | . | . | . | . | . | . | . | . | . | . | . | . | . | . | . | . | . | . | . | [2970] |
| NY99_USA_2005 | A | H | L | R | G | E | C | H | T | C | I | Y | N | M | M | G | K | R | E | K | K | P | G | E | F | G | K | A | K | G | [3000] |
| ATH002316_Kenya_2012 | . | . | . | . | . | . | . | . | . | . | . | . | . | . | . | . | . | . | . | . | . | . | . | . | . | . | . | . | . | . | [3000] |
| NY99_USA_2005 | S | R | A | I | W | F | M | W | L | G | A | R | F | L | E | F | E | A | L | G | F | L | N | E | D | H | W | L | G | R | [3030] |
| ATH002316_Kenya_2012 | . | . | . | . | . | . | . | . | . | . | . | . | . | . | . | . | . | . | . | . | . | . | . | . | . | . | . | . | . | . | [3030] |
| NY99_USA_2005 | K | N | S | G | G | G | V | E | G | L | G | L | Q | K | L | G | Y | I | L | R | E | V | G | T | R | P | G | G | K | I | [3060] |
| ATH002316_Kenya_2012 | . | . | . | . | . | . | . | . | . | . | . | . | . | . | . | . | . | . | . | . | . | . | . | . | . | . | . | . | . | . | [3060] |
| NY99_USA_2005 | Y | A | D | D | T | A | G | W | D | T | R | I | T | R | A | D | L | E | N | E | A | K | V | L | E | L | L | D | G | E | [3090] |
| ATH002316_Kenya_2012 | . | . | . | . | . | . | . | . | . | . | . | . | . | . | . | . | . | . | . | . | . | . | . | . | . | . | . | . | . | . | [3090] |
| NY99_USA_2005 | H | R | R | L | A | R | A | I | I | E | L | T | Y | R | H | K | V | V | K | V | M | R | P | A | A | D | G | R | T | V | [3120] |
| ATH002316_Kenya_2012 | . | . | . | . | . | . | . | . | . | . | . | . | . | . | . | . | . | . | . | . | . | . | . | . | . | . | . | . | . | . | [3120] |
| NY99_USA_2005 | M | D | V | I | S | R | E | D | Q | R | G | S | G | Q | V | V | T | Y | A | L | N | T | F | T | N | L | A | V | Q | L | [3150] |
| ATH002316_Kenya_2012 | . | . | . | . | . | . | . | . | . | . | . | . | . | . | . | . | . | . | . | . | . | . | . | . | . | . | . | . | . | . | [3150] |
| NY99_USA_2005 | V | R | M | M | E | G | E | G | V | I | G | P | D | D | V | E | K | L | T | K | G | K | G | P | K | V | R | T | W | L | [3180] |
| ATH002316_Kenya_2012 | . | . | . | . | . | . | . | . | . | . | . | . | . | . | . | . | . | . | . | . | . | . | . | . | . | . | . | . | . | . | [3180] |
| NY99_USA_2005 | F | E | N | G | E | E | R | L | S | R | M | A | V | S | G | D | D | C | V | V | K | P | L | D | D | R | F | A | T | S | [3210] |

| | | | | | | | | | | | | | | | | | | | | | | | | | | | | | | | |
|---|---|---|---|---|---|---|---|---|---|---|---|---|---|---|---|---|---|---|---|---|---|---|---|---|---|---|---|---|---|---|---|
| ATH002316_Kenya_2012 | . | . | . | . | . | . | . | . | . | . | . | . | . | . | . | . | . | . | . | . | . | . | . | . | . | . | . | . | . | . | [3210] |
| NY99_USA_2005 | L | H | F | L | N | A | M | S | K | V | R | K | D | I | Q | E | W | K | P | S | T | G | W | Y | D | W | Q | Q | V | P | [3240] |
| ATH002316_Kenya_2012 | . | . | . | . | . | . | . | . | . | . | . | . | . | . | . | . | . | . | . | . | . | . | . | . | . | . | . | . | . | . | [3240] |
| NY99_USA_2005 | F | C | S | N | H | F | T | E | L | I | M | K | D | G | R | T | L | V | V | P | C | R | G | Q | D | E | L | V | G | R | [3270] |
| ATH002316_Kenya_2012 | . | . | . | . | . | . | . | . | . | . | . | . | . | . | . | . | . | . | . | . | . | . | . | . | . | . | . | . | . | . | [3270] |
| NY99_USA_2005 | A | R | I | S | P | G | A | G | W | N | V | R | D | T | A | C | L | A | K | S | Y | A | Q | M | W | L | L | L | Y | F | [3300] |
| ATH002316_Kenya_2012 | . | . | . | . | . | . | . | . | . | . | . | . | . | . | . | . | . | . | . | . | . | . | . | . | . | . | . | . | . | . | [3300] |
| NY99_USA_2005 | H | R | R | D | L | R | L | M | A | N | A | I | C | S | A | V | P | V | N | W | V | P | T | G | R | T | T | W | S | I | [3330] |
| ATH002316_Kenya_2012 | . | . | . | . | . | . | . | . | . | . | . | . | . | . | . | . | . | . | . | . | . | . | . | . | . | . | . | . | . | . | [3330] |
| NY99_USA_2005 | H | A | G | G | E | W | M | T | T | E | D | M | L | E | V | W | N | R | V | W | I | E | E | N | E | W | M | E | D | K | [3360] |
| ATH002316_Kenya_2012 | . | . | . | . | . | . | . | . | . | . | . | . | . | . | . | . | . | . | . | . | . | . | . | . | . | . | I | . | . | . | [3360] |
| NY99_USA_2005 | T | P | V | E | K | W | S | D | V | P | Y | S | G | K | R | E | D | I | W | C | G | S | L | I | G | T | R | A | R | A | [3390] |
| ATH002316_Kenya_2012 | . | . | . | . | . | . | . | . | . | . | . | . | . | . | . | T | . | . | . | . | . | . | . | . | . | . | . | . | . | . | [3390] |
| NY99_USA_2005 | T | W | A | E | N | I | Q | V | A | I | N | Q | V | R | A | I | I | G | D | E | K | Y | V | D | Y | M | S | S | L | K | [3420] |
| ATH002316_Kenya_2012 | . | . | . | . | . | . | . | . | . | . | . | . | . | . | . | . | . | . | . | . | . | . | . | . | . | . | . | . | . | . | [3420] |
| NY99_USA_2005 | R | Y | E | D | T | T | L | V | E | D | T | V | L | * | [3434] | | | | | | | | | | | | | | | | |
| ATH002316_Kenya_2012 | . | . | . | . | . | . | . | . | . | . | . | . | . | . | [3434] | | | | | | | | | | | | | | | | |

Sequence alignment of the WNV polyprotein gene that corresponds to the structural (C, E and M) and non-structural (1, 2A, 2B, 3, 4A, 4B and 5) proteins sequences of the Kenyan WNV isolate (ATH002316) and the reference strain (NC_009942.1). Region sequenced; position 1 to 3434. Study isolate highlighted in red.
